# Benchmarking broad-spectrum antibiotic use in older adult pneumonia inpatients: a risk-adjusted smoothed observed-to-expected ratio approach

**DOI:** 10.1017/ice.2025.5

**Published:** 2025-04

**Authors:** Abbas Khatoun, Noriko Sasaki, Susumu Kunisawa, Kiyohide Fushimi, Yuichi Imanaka

**Affiliations:** 1 Department of Healthcare Economics and Quality Management, Graduate School of Medicine, Kyoto University, Kyoto, Japan; 2 Department of Health Policy and Informatics, Graduate School of Medical and Dental Sciences, Tokyo Medical and Dental University, Tokyo, Japan; 3 Department of Health Security System, Centre for Health Security, Graduate School of Medicine, Kyoto University, Kyoto, Japan

**Keywords:** benchmarking antibiotic use, risk adjustment, smoothed observed-to-expected ratio, broad-spectrum antibiotics, antimicrobial stewardship

## Abstract

**Objective:**

Antimicrobial resistance is increased by antibiotic overuse, so it’s crucial for stewardship programs to monitor and control their use. Pneumonia, particularly prevalent among older adults in Japan, is requiring higher rates of medical treatment. This study aimed to develop an improved method for benchmarking broad-spectrum antibiotic use in the empiric treatment of pneumonia in older adult inpatients by applying the “smoothed” observed-to-expected (O/E) ratio which adjusts for hospital-level variations and minimizes the effect of extreme values.

**Methods:**

Using nationwide data from the Diagnosis Procedure Combination research group, pneumonia patients between April 1^st^ 2018 and March 31^st^ 2020 were analyzed. The primary outcome was the smoothed O/E ratio of the broad-spectrum antibiotic use for hospitals. It was calculated from the predicted values of broad-spectrum antibiotic use that were obtained through multilevel logistic regression using patient characteristics as predictors from data clustered by hospitals. The analysis investigated the risk-adjusted use of broad-spectrum antibiotics among hospitals.

**Results:**

A total of 244,747 patients from 958 hospitals were included, with a mean age of 81 (±8.30) years. The proportion of broad-spectrum antibiotic use was 35.3% (n = 86,316). The prediction model showed a C-statistic of 0.722. There was a noticeable variation in the O/E ratio among hospitals with values ranging from 0.13 (95% CI: 0.09–0.20) to 2.81 (95% CI: 2.64–2.97).

**Conclusions:**

Using a risk-adjusted smoothed O/E ratio, we assessed the use of broad-spectrum antibiotics across hospitals, identifying those with high O/E ratios that may indicate a need for improvement.

## Introduction

The World Health Organization (WHO) classified antimicrobial resistance (AMR) as one of the ten main global health issues to be tracked in 2021.^1^ High use of antibiotics, use of multiple broad-spectrum antibiotics, and lack of proper antimicrobial stewardship are considered some of the foremost factors responsible for the increase in AMR.^2^ Hospitals are an example of a setting where antibiotic usage is showing evident increase, and the recent years have shown a significant increase in the use of broad-spectrum antibiotics such as vancomycin, carbapenems, and fourth-generation cephalosporins.^3,4^ The proper antimicrobial stewardship of broad-spectrum antibiotics has shown to reduce their unnecessary overuse.^5^

Pneumonia is a major infectious disease with higher incidence, mortality, and rates of antimicrobial treatment in older adults.^6,7^ It’s often treated with broad-spectrum antibiotics including anti-MRSA (Methicillin-resistant Staphylococcus aureus) and antipseudomonal agents.^8^ However, research findings and guidelines emphasize the significance of appropriate antimicrobial therapies, warning that the liberal use of antimicrobial combinations will eventually generate untreatable resistant infections.^9^ In Japan, pneumonia is growing in significance due to the ageing population.^6^ As it’s primarily affecting those aged over 64, pneumonia is requiring higher rates of medical treatment and ranking as the fifth leading cause of death, making its management increasingly important.^6,10^

Quantifying antibiotic use is crucial for antimicrobial stewardship programs. Although one definitive metric or method has yet to be determined, quantitative evaluation can aid the development of stewardship programs for monitoring and promoting their responsible use in hospitals.^11–13^ Since comparisons across hospitals using unadjusted metrics are discouraged, various risk adjustment approaches for antimicrobial use have been developed, incorporating patient or hospital-related predictors in regression modeling.^14,15^ An example of that is the observed-to-expected (O/E) ratio, which has served as a quality indicator in the past for various measures related to inpatient care, including antimicrobial use.^12,15–17^ While the basic O/E ratio is useful, it is still vulnerable to sample variation. Thus, smoothing techniques, such as those applied by the Agency for Healthcare Research and Quality, can help stabilize estimates.^18^ Therefore, this study aimed to develop an improved method for benchmarking broad-spectrum antibiotic use in the empiric treatment of pneumonia in older adult inpatients by applying the smoothed O/E ratio and adjusting for varying patient risk profiles across hospitals.

## Methods

### Data source

Patient and hospital data were obtained from the Diagnosis Procedure Combination (DPC) research group’s database, funded by the Ministry of Health, Labour and Welfare (MHLW). DPC system is a measuring tool intended to standardize Japanese medical care by making acute patient care transparent.^19^ The database includes survey data defined by MHLW, containing information from acute care hospitals, such as patient clinical information and disease classifications based on the 10^th^ Revision of the International Classification of Diseases (ICD-10), as well as claims data.^19,20^ Acute care hospitals periodically and officially submit DPC data to MHLW, and copies of this data were provided to the DPC research group.

### Study population

We selected patients hospitalized and discharged between April 1^st^, 2018, and March 31^st^, 2020. Patients were included in the study if they were over 64 years old, admitted for pneumonia, and received antibiotic treatment within the first 48 hours of admission. Pneumonia diagnosis, recorded as both the admission-precipitating diagnosis and the most resource-consuming diagnosis, was based on the ICD-10 codes J13, J14, J15.x, J16.x, J17.x, and J18.x. However, patients who had undergone surgery prior to treatment were excluded, as this is uncommon in pneumonia admissions, and they were considered as perioperative prophylaxis cases. Patients were also excluded if they had missing data related to the variables needed in risk adjustment. In addition, hospitals with less than a total of 100 patient cases were excluded.

### Outcome

The primary outcome in this study was the smoothed O/E ratio of the use of broad-spectrum antibiotics across the hospitals. Broad-spectrum antibiotic use at the patient level was quantified as the exposure to these antibiotics during empiric treatment (ie, whether or not the patient received them during these 48 hours).^21^ In this study, broad-spectrum antibiotics were defined as antibiotics reserved for patients with risk of MDR pathogens, according to the Japanese Association for Infectious Diseases/Japanese Society of Chemotherapy guideline for pneumonia treatment.^22^ Additionally, some agents not in the guideline were considered as antibiotics reserved for last-line treatment, following the WHO’s “AWaRe” classification.^23^ Consequently, the broad-spectrum antibiotic agents in our study were: Aztreonam, Biapenem, Cefepime, Cefozopran, Ceftolozane-Tazobactam, Ciprofloxacin, Colistin, Daptomycin, Doripenem, Faropenem, Fosfomycin, Imipenem, Lascufloxacin, Linezolid, Meropenem, Minocycline, Panipenem, Pazufloxacin, Piperacillin-Tazobactam, Polymyxin B, Prulifloxacin, Tebipenem, Tedizolid, Teicoplanin, Tigecycline, Vancomycin. It is worth mentioning that the definition of broad-spectrum antibiotics could differ for some agents depending on the resources, so our definition applies to this study.

### O/E ratio and smoothed O/E ratio

The O/E ratio, which follows the concept of the standardized mortality ratio, is usually calculated by dividing the observed value, as the numerator, by the expected value, which is obtained through a prediction model, as the denominator.^24^

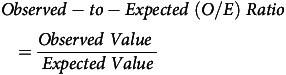

As a simplified general concept, a hospital’s O/E value below 1 will indicate that the broad-spectrum antibiotic use is lower than expected, while a value above 1 is higher.

Mohammed *et al.* differentiated between several methods of deriving the standardized mortality O/E ratio. Including the standard one above, he introduced another which can be referred to as the “smoothed” O/E ratio. It differs than the standard O/E ratio by utilizing the smoothed observed values as the numerator instead of the crude (raw) observed values.^24^

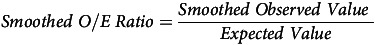

The smoothed observed value, which is estimated using a multilevel model with a random intercept, shrinks sample variation in the crude observed values.

### Baseline variables and model development

A multilevel prediction model is needed to calculate the smoothed O/E ratio of broad-spectrum antibiotic use among hospitals. The model can be represented as:

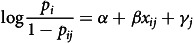


*p*_*ij*_ is the probability of broad-spectrum antibiotic use for a certain patient (*i*) treated at a certain hospital (*j*), *x*_*ij*_ is the vector of risk adjustment variables, α represents the model intercept, β is the coefficient for *x*_*ij*_, and γ_j_ is the random intercept.^24^

Predictors for our model included patient-related variables reported in previous literature as risk factors for resistance in pneumonia, as well as disease severity criteria.^22,25–29^ The disease severity variables were based on the A-DROP (Age, Dehydration, Respiratory failure, Orientation disturbance, Blood pressure) score, a modified version of the CURB-65 (Confusion, Uremia, Respiratory rate, Blood pressure, Age≥65) score for pneumonia severity, proposed by the Japanese Respiratory Society, with predictive power similar to that of the CURB-65.^28^ Afterwards, the following variables were selected as predictors: age, sex, ICD-10 codes, variables composing A-DROP score (blood urea nitrogen (BUN), oxygen saturation (SpO_2_), orientation disturbance, systolic blood pressure), immunodeficiency, previous hospitalization within the last 90 days, admission from home, admission from nursing home, intensive care unit (ICU) admission, being on mechanical ventilator, tube feeding, and various comorbidities on admission (congestive heart failure, hypertension, coronary obstructive pulmonary disease, diabetes, dementia, cerebrovascular disease, renal disease, rheumatic disease, cancer, pulmonary circulation disorder).

A multilevel logistic prediction model, with the hospital identifiers as the random intercept, was developed based on the previously mentioned predictors. The random intercept allows the calculation of the smoothed O/E ratio and accommodates the clustering effect of the hospitals in our dataset.^30^ Since we included multiple predictors in our model, we recognized the potential for multicollinearity. By checking the variance inflation factor, we found that most predictors displayed low levels, with only a couple showing slightly moderate levels, confirming that multicollinearity wasn’t a significant concern. Furthermore, following the recommendations of the “Transparent Reporting of a Multivariable Prediction Model for Individual Prognosis or Diagnosis” (TRIPOD) statement, we performed validation for our prediction model. The data was split non-randomly by fiscal years (FY) into a training dataset (FY2018: from April 1^st^, 2018 till March 31^st^, 2019) and a validation dataset (FY2019: from April 1^st^, 2019 till March 31^st^, 2020).^31^ Afterwards, model validation was performed by assessing and comparing performances in the training, validation, and overall datasets. The parameters for the performance assessment included: concordance statistic (C-statistic), the means of the observed use and the predicted probabilities, the calibration slope, and the accuracy.^32^

### Statistical analysis

We used our validated multilevel prediction model to obtain the risk-adjusted broad-spectrum antibiotic use and calculate the smoothed O/E ratios for each hospital as follows:

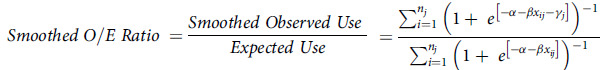




The numerator is the sum of the probabilities of broad-spectrum antibiotic use from the multilevel model with the random intercept, while the denominator reflects the sum of the predictions from the same model without the random intercept. The confidence intervals (CIs) for the obtained Smoothed O/E ratios were generated through bootstrapping with 500 iterations, providing reliable estimates of variability.

All the statistical analyses were performed using R statistical software version 4.1.1 (R Foundation for Statistical Computing, Vienna, Austria), and the “lme4” package (ver 1.1.31) was used for fitting multilevel models. A *P*-value < .05 was considered statistically significant.

### Ethics approval

The study was conducted in accordance with the “Ethical Guidelines for Medical and Health Research Involving Human Subjects” issued by the Ministry of Education, Culture, Sports, Science and Technology, the Ministry of Health, Labour, and Welfare, and the Ministry of Economy, Trade, and Industry. Moreover, it utilized anonymous data, and the need for informed consent from the study participants was waived by disclosing research information to the public. It was also approved by the ethics committee of the Graduate School of Medicine, Kyoto University (R0135).

## Results

**Figure 1.**
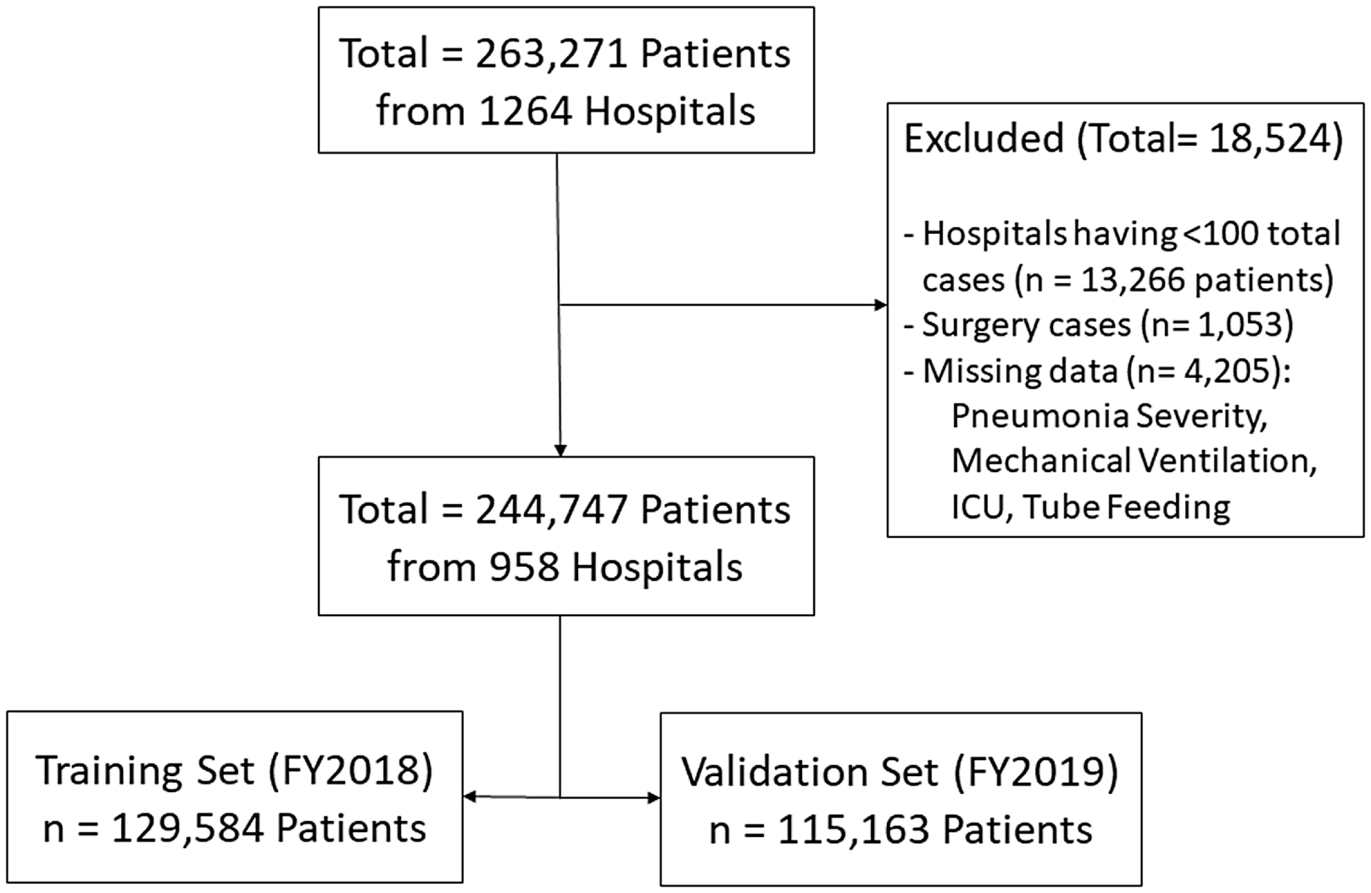
Flow diagram showing the selected dataset and the model validation subsets. Abbreviations: ICU, intensive care unit; FY, fiscal year.

A total of 263,271 patients from 1,264 hospitals who met the inclusion criteria were extracted from the database. Figure 1 shows the selection process. Hospitals with fewer than 100 total patient cases were excluded, as some hospitals had very low numbers of total cases, while our model included 24 predictors. There was no notable difference in the data characteristics before and after excluding these cases (Supplementary Table). Eventually, 244,747 patients from 958 hospitals were analyzed. Furthermore, the training and validation datasets resulting from splitting the data consisted of 129,584 and 115,163 patients, respectively.

Table 1 summarizes the baseline characteristics of the study population. The mean age was 81 (± 8.30) years, and 41.7% of the patients were female. Moreover, among all patients, 35.3% (n = 86,316) showed use of broad-spectrum antibiotics. In addition, the proportion of use of broad-spectrum antibiotics was higher among variables such as previous hospitalization in the last 90 days (44.4%), being on mechanical ventilator (62.5%), and being in the ICU (51.1%).

**Table 1.** Baseline patient characteristics in the dataset

Variable	n (%) or mean (± SD)
n	244,747 (100)
Age, years	81 (± 8.30)
Age categories	
65–74 years old	51,600 (21.08)
75–84 years old	92,329 (37.72)
85 years old & above	100,818 (41.19)
Sex	
Male	142,610 (58.27)
Female	102,137 (41.73)
A-DROP score variables	
BUN > 21 mg/dl	108,181 (44.20)
SpO_2_ ≤ 90%	102,953 (42.07)
Loss of consciousness	35,198 (14.38)
Systolic blood pressure < 90 mmHg	12,824 (5.24)
Immunodeficiency	45,481 (18.58)
ICD-10 codes	
J13 (streptococcus pneumonia)	20,898 (8.54)
J14 (haemophilus influenza)	3,776 (1.54)
J15.x (bacterial pneumonia, not classified)	95,630 (39.07)
J16.x (other infectious agents, not classified)	207 (0.08)
J17.x (Pneumonia in diseases classified elsewhere)	1,096 (0.45)
J18.x (unknown pathogen)	123,140 (50.31)
Previous hospitalization in the last 90 days	44,102 (18.02)
Admission from home	200,645 (81.98)
Admission from nursing home	37,750 (15.42)
Admitted to the ICU	16,814 (6.87)
On mechanical ventilator	6,010 (2.46)
Receiving tube feeding	1,309 (0.53)
Comorbidities on admission	
Congestive heart failure	54,723 (22.36)
Hypertension	78,350 (32.01)
Coronary obstructive pulmonary disease	54,095 (22.10)
Diabetes	48,746 (19.92)
Dementia	35,143 (14.36)
Cerebrovascular disease	27,971 (11.43)
Renal disease	17,225 (7.04)
Rheumatic disease	9,130 (3.73)
Cancer	30,761 (12.57)
Pulmonary circulation disorder	1,532 (0.63)

Abbreviations: BUN, blood urea nitrogen; SpO_2_, saturation of peripheral oxygen; ICD, international classification of diseases; ICU, intensive care unit.

Table 2 sums up the model’s performance for the training set, validation set, and the combined full dataset through the previously mentioned performance parameters. There was minimal change in the performance parameters across the three sets.

**Table 2.** Model validation and performance

Performance parameters	Training set (n = 129,584)	Validation set (n = 115,163)	Combined data (n = 244,747)
C-statistic (area under the curve)	0.727	0.715	0.722
Mean of the observed use vs mean of the predicted probabilities	0.36 vs 0.36	0.35 vs 0.36	0.35 vs 0.36
Calibration slope	1.03	0.95	0.99
Accuracy	67.4%	66.5%	67.0%

**Figure 2.**
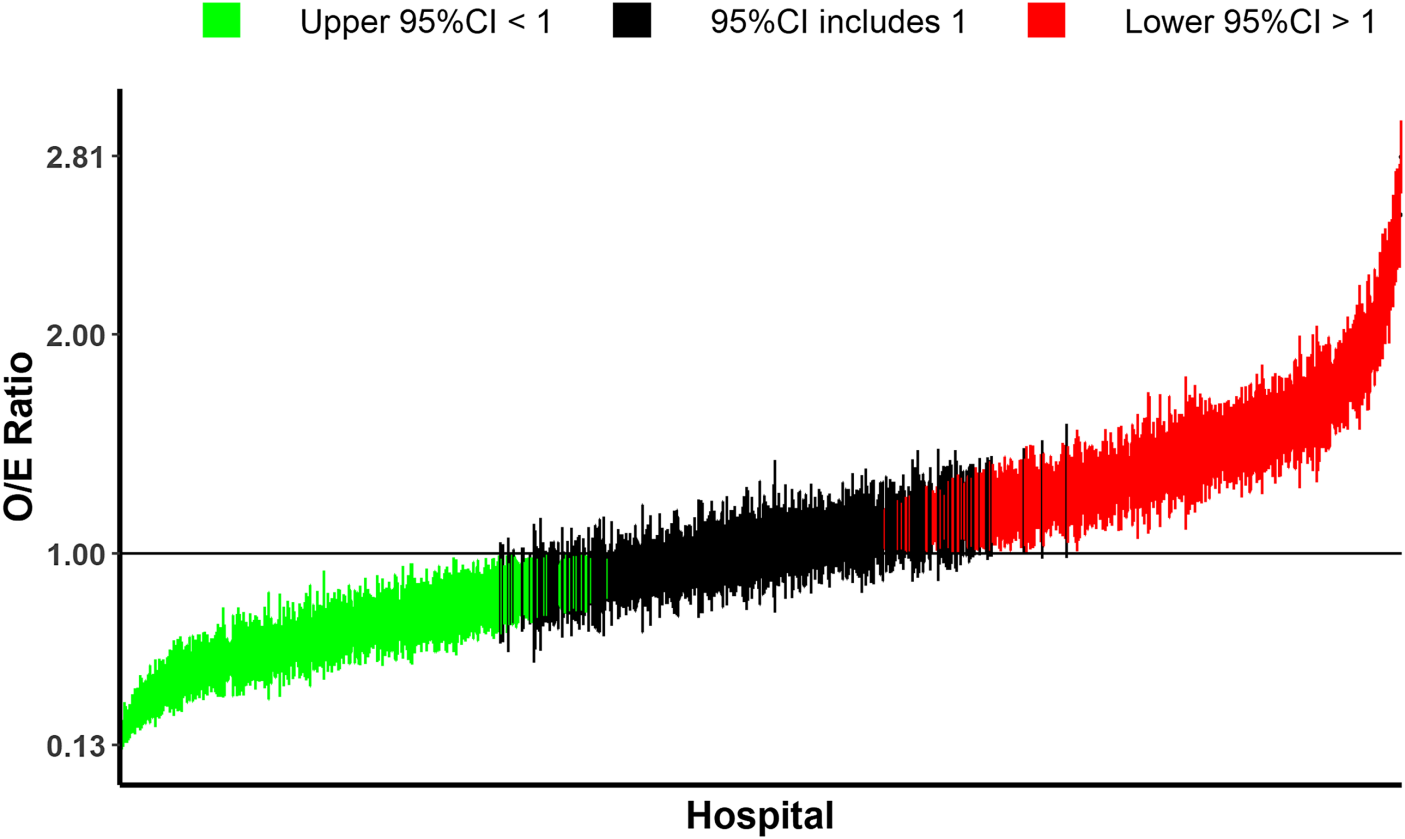
Smoothed observed-to-expected (O/E) ratios of the use of broad-spectrum antibiotics among the hospitals. The dots and bars indicate the Smoothed O/E ratios and the 95% confidence intervals (CI), respectively. The left part represents hospitals with an upper 95% CI of the O/E ratio that is less than 1 (n = 312), while the right part represents hospitals showing a lower 95% CI of the O/E ratio greater than 1 (n = 331). Consequently, the middle black part represents the hospitals of which 95% CI includes 1 (n = 315).

The C-statistics of our multilevel model were 0.722 and 0.632 with and without the random intercept, respectively. Figure 2 shows the smoothed O/E ratios of the use of broad-spectrum antibiotics as empiric therapy and their confidence intervals (CIs) across hospitals (sorted in ascending order). The graph demonstrates variability among the hospitals, with the lowest and highest ratio values being 0.13 (95% CI: 0.09–0.20) and 2.81 (95% CI: 2.64–2.97), respectively. Among the 958 hospitals, 312 had an upper 95% CI of the O/E ratio less than 1, indicating that the use of broad-spectrum antibiotics was less than the risk-adjusted average, while 331 hospitals showed a lower 95% CI greater than 1, indicating that the use was higher. Consequently, 315 hospitals had CIs crossing 1. Overall, there was notable variation in the smoothed O/E ratios among the hospitals.

## Discussion

Using a nationwide inpatient database, we quantitatively benchmarked the use of broad-spectrum antibiotics in empiric therapy for older adult pneumonia inpatients through a risk-adjusted smoothed O/E ratio. Results showed wide variations among hospitals, and our method enabled the assessment of how much hospitals are using these antibiotics according to the risk-adjusted average. Subsequently, we were able to indicate which hospitals show a high ratio of broad-spectrum antibiotic use without being affected by patient characteristics and hospital differences.

Our study showed considerable variation among hospitals in the use of broad-spectrum antibiotics after performing risk adjustment. These findings align with previous studies that have also used a diagnosis-related system to establish risk-adjusted O/E ratios for benchmarking the use of various antibiotics.^12,16,17^ In contrast to Polk *et al.*, who used indirect standardization, and Tan *et al.*, who utilized facility-level data and single-level regression, our study employed multilevel regression analysis with patient-level data and a random effect for facilities. This approach provides more detailed information for risk adjustment and appropriately accounts for the clustered nature of the patient data within hospitals, enhancing the accuracy of our estimates and improving comparisons among facilities.

Although Yu *et al.* also employed a multilevel model in one of their approaches, a key distinction in our study is the application of the smoothed O/E ratio rather than the standard O/E ratio. The smoothed O/E ratio has not been previously applied for benchmarking antimicrobial use, making its inclusion in this study a novel approach. The numerator of the smoothed O/E ratio (ie, Smoothed Observed Use) is a refined model-based estimate that minimizes the influence of extreme values and sample variation in the crude observed data. This approach enhances estimation and assessment in our study by providing a more reliable measurement. Moreover, our graph visualization of the ratio and CIs for each hospital offers a clearer indication of variability and statistical uncertainty compared to other studies, thereby enhancing the transparency of our benchmarking process.

While the previously mentioned studies used various quantity metrics for antibiotic use, such as days of therapy and defined daily dose (DDD), we used patient exposure as our metric.^12,16,17^ Although the WHO recommends the defined daily dose (DDD) for measuring antibiotic consumption, there’s no gold standard for the magnitude of antimicrobial use, and DDD alone doesn’t always show a realistic picture in practice, as different metrics reflect different attributes of use.^12,33,34^ Since we are benchmarking the use of broad-spectrum antibiotics in empiric therapy, ie the first 48 hours of admission, the metric of exposure was suitable for our scope, and it was selected from a set of evidence-based and validated quantity metrics for antibiotic use.^21^ Nonetheless, we acknowledge that the duration of therapy is another important factor that should be considered or can be reflected using other metrics.

The inter-hospital variation in our study could be attributed to other factors such as resistance rates. However, since hospitals across Japan don’t drastically differ in rates of resistant pneumonia pathogens, the variation could be due to other modifiable factors.^35^ Thus, our method can aid in reducing the use regardless. Moreover, a previous study, aimed to identify patient and hospital factors that may contribute to differences in antibiotic use, has pointed out that university-affiliated hospitals showed a lower level of antibiotic unitization than other institutions.^36^ Similarly, in our study, when we calculated the smoothed O/E ratio among hospitals grouped by teaching status, the group of non-teaching hospitals showed a slightly high ratio (1.22) compared to the teaching hospitals (0.98). It is worth noting that this is merely an observation for now, but further investigation could lead to more reliable inferences.

Our findings identified hospitals with high O/E ratios of broad-spectrum antibiotic use that may require further revision. By providing findings as feedback, hospitals can utilize such data as a resource for identifying potential needs for improvement and aligning with antimicrobial stewardship objectives. By providing more reliable and stable estimates than the standard O/E ratio, the smoothed O/E ratio enhances the accuracy of hospital comparisons, supporting better-informed decisions. Additionally, these findings can also assist policymakers and healthcare providers in understanding the situation regarding the use of these antibiotics for older adult pneumonia patients, guiding efforts to decrease their use in hospitals. Furthermore, this study may serve as a benchmark for future investigations of antibiotic use in hospitals, allowing subsequent research to explore areas of improvement in identified hospitals, to improve antimicrobial stewardship. It can also serve as a guide for future studies focusing on other types of infections.

This study was strengthened by the large sample size across multiple hospitals. Additionally, considering the hospital random intercept and smoothed O/E ratio provided an advantage for our approach and assessment over the standard O/E ratio. Moreover, presenting the 95% confidence intervals derived from bootstrapping demonstrated the uncertainty in our estimations.

Our study also had some limitations. First, the clinical appropriateness of the use of broad-spectrum antibiotics for patients individually was not examined. While that is important in clinical practice, our analysis focused on risk-adjusted benchmarking rather than clinical appropriateness. In addition, data that would allow for this direct assessment, such as microbiology results, was not available in DPC. Second, this study only included older adults treated for pneumonia from Japanese acute care hospitals. Although this indicates that the findings cannot be generalized to full populations or other infections, they do, however, emphasize a significant aspect of an important infectious disease primarily affecting that age group. The study’s purpose was not to benchmark for a full population, but rather to focus on pneumonia as a main infection with a high volume of antibiotic use.

In conclusion, we were able to demonstrate variability in broad-spectrum antibiotic use in the empiric treatment of pneumonia in older adults among hospitals through a risk-adjusted smoothed O/E ratio that considers patient-level risk factors and random variations. Additionally, our method identified the hospitals with high O/E ratios, where reflecting on practices may lead to beneficial changes.

## Supporting information

Khatoun et al. supplementary material Khatoun et al. supplementary material

## Supplementary material

The supplementary material for this article can be found at https://doi.org/10.1017/ice.2025.5

## Manuscript preparation

We thank Dr. Jung-ho Shin for his valuable support during the study.
