# Supplementary material for: Benchmarking broad-spectrum antibiotic use in older adult pneumonia inpatients: a risk-adjusted smoothed observed-to-expected ratio approach
Source: Infect Control Hosp Epidemiol. 2025 Feb 17;46(4):410–5. doi: 10.1017/ice.2025.5 (PMC12015619; doi:10.1017/ice.2025.5)
Supplement: Khatoun et al. supplementary material [file S0899823X25000054sup001.pdf]

Supplementary Material

Supplementary Table. Comparison of the datasets before and after excluding the hospitals with less than 100 total cases per hospital (Note: surgery cases and missing data cases are already excluded from the totals).

Variable	n (%) or mean (± SD)	
n	258,013 (100)	244,747 (100)
Age, years	81 (± 8.32)	81 (± 8.30)
Age Categories		
65-74 years old	54,396 (21.08)	51,600 (21.08)
75-84 years old	96,942 (37.57)	92,329 (37.72)
85 years old & above	106,675 (41.34)	100,818 (41.19)
Sex		
Male	149,978 (58.13)	142,610 (58.27)
Female	108,035 (41.87)	102,137 (41.73)
A-DROP Score Variables		
BUN > 21 mg/dL	113,883 (44.14)	108,181 (44.20)
SpO2 ≤ 90%	107,519 (41.67)	102,953 (42.07)
Loss of Consciousness	36,822 (14.27)	35,198 (14.38)
SBP < 90 mmHg	13,567 (5.26)	12,824 (5.24)
ICD-10 Codes		
J13	21,819 (8.46)	20,898 (8.54)
J14	3,939 (1.53)	3,776 (1.54)
J15.x	100,188 (38.83)	95,630 (39.07)
J16.x	219 (0.08)	207 (0.08)
J17.x	1,171 (0.45)	1,096 (0.45)
J18.x	130,677 (50.65)	123,140 (50.31)
Immunodeficiency	47,614 (18.45)	45,481 (18.58)
Previous Hospitalization	46,545 (18.04)	44,102 (18.02)

from Home	210,657 (81.65)	200,645 (81.98)
from Nursing Home	40,335 (15.63)	37,750 (15.42)
Admitted in the ICU	17,151 (6.65)	16,814 (6.87)
On Mechanical Ventilator	6,180 (2.40)	6,010 (2.46)
Receiving Tube Feeding	1,443 (0.56)	1,309 (0.53)
Comorbidities		
Congestive Heart Failure	57,485 (22.28)	54,723 (22.36)
Hypertension	82,750 (32.07)	78,350 (32.01)
COPD	56,497 (21.90)	54,095 (22.10)
Diabetes	51,056 (19.79)	48,746 (19.92)
Dementia	37,139 (14.39)	35,143 (14.36)
Cerebrovascular Disease	29,563 (11.46)	27,971 (11.43)
Renal Disease	18,106 (7.02)	17,225 (7.04)
Rheumatic Disease	9,572 (3.71)	9,130 (3.73)
Cancer	32,141 (12.46)	30,761 (12.57)
Pulmonary Circulation Disorder	1,593 (0.62)	1,532 (0.63)
